# In Vivo Expansion of Co-Transplanted T Cells Impacts on Tumor Re-Initiating Activity of Human Acute Myeloid Leukemia in NSG Mice

**DOI:** 10.1371/journal.pone.0060680

**Published:** 2013-04-09

**Authors:** Malte von Bonin, Martin Wermke, Kadriye Nehir Cosgun, Christian Thiede, Martin Bornhauser, Gerard Wagemaker, Claudia Waskow

**Affiliations:** 1 Regeneration in Hematopoiesis, Center for Regenerative Therapies Dresden – CRTD, DFG Research Center and Cluster of Excellence, Technische Universität Dresden, Dresden, Germany; 2 Medizinische Klinik und Poliklinik 1, Universitätsklinikum Carl-Gustav-Carus, Technische Universität Dresden, Dresden, Germany; 3 Department of Hematology, Erasmus University Medical Center, Rotterdam, The Netherlands; RWTH Aachen University Medical School, Germany

## Abstract

Human cells from acute myeloid leukemia (AML) patients are frequently transplanted into immune-compromised mouse strains to provide an *in vivo* environment for studies on the biology of the disease. Since frequencies of leukemia re-initiating cells are low and a unique cell surface phenotype that includes all tumor re-initiating activity remains unknown, the underlying mechanisms leading to limitations in the xenotransplantation assay need to be understood and overcome to obtain robust engraftment of AML-containing samples. We report here that in the NSG xenotransplantation assay, the large majority of mononucleated cells from patients with AML fail to establish a reproducible myeloid engraftment despite high donor chimerism. Instead, donor-derived cells mainly consist of polyclonal disease-unrelated expanded co-transplanted human T lymphocytes that induce xenogeneic graft versus host disease and mask the engraftment of human AML in mice. Engraftment of mainly myeloid cell types can be enforced by the prevention of T cell expansion through the depletion of lymphocytes from the graft prior transplantation.

## Introduction

Xenotransplantation has become an indispensable tool for the study of human stem cell biology *in vivo*, and the continuous development of novel recipient mouse strains documents the high interest and use of this technology [1]–[5]. Using mice as surrogate organisms has the advantage that adult human stem cells, including hematopoietic stem cells and cancer stem cells can be modulated and effects analyzed *in vivo*. In fact, cancer stem cells are defined by their capacity to re-initiate human tumors after transplantation into mice [6], but variations in the transplantation settings and the use of different recipient mouse strains resulted in inconsistent results on the frequency and phenotype of this cell type [7]–[11] pointing at the urge to determine the frequency of tumor re-initiating activity in non-fractionated material in a robust xenotransplantation assay. Furthermore, antibody coating of human cells might influence the engraftment capability and bias the identification of stem cells according to their surface markers [10].

Acute myeloid leukemia (AML) is a rare, heterogeneous malignancy that originates from hematopoietic cells [12]. Treatment therapy usually results in complete remission in the majority of patients younger than 65. However, most patients will relapse and only 30% of adults diagnosed with AML are expected to survive three or more years [12]. Relapse of the disease may be explained by the outgrowth of a small fraction of tumor cells that survived therapy and that have the capacity to re-initiate the tumor [13]. It has been suggested that these surviving tumor cells represent the cancer stem cell fraction that re-initiates the tumor growth in mice [13]. The concept that cancer stem cells contain all tumor re-initiating activity is clinically of major importance because of its potential impact on therapy [14]–[20]. However, the cancer stem cell concept was recently challenged by experiments that evidenced tumor re-initiating activity outside the cancer stem cell fraction in AML material [7]–[10]. Furthermore, engraftment capacity of human cells can be negatively affected by antibody coating [10]. Thus, to analyze tumor re-initiation activity present in AML samples, it is necessary to transplant non-fractionated, non-marked tumor cells, and to score AML engraftment by analyzing donor-derived diseased cells.

## Results

### Factors Altering the Engraftment of Human AML in NSG Mice

**Figure 1:**
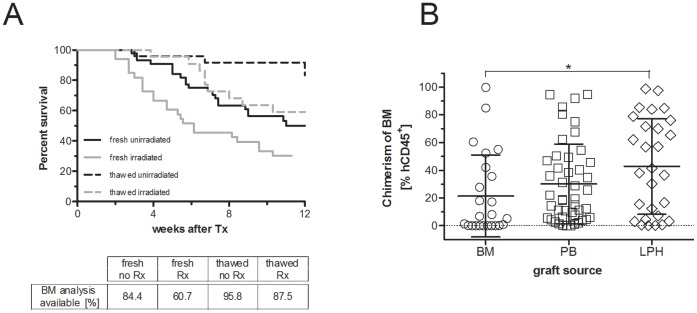
Sublethal irradiation and fresh donor cells reduces survival in NSG mice transplanted with AML-MNCs. (A) Kaplan-Meyer plot of mice that were transplanted with MNCs of AML patients. NSG mice were transplanted with freshly isolated MNCs with (fresh/irradiated; grey solid line) and without (fresh/non-conditioned; black solid line) previous conditioning, or thawed MNCs with (thawed/irradiated; grey dotted line) or without (thawed/non-conditioned; black dotted line) sublethal irradiation. The percentage of mice transplanted and available for bone marrow analysis is indicated below graph (BM analysis available). Using fresh donor cells and irradiation conditioning shortened the life span of the recipient mice (fresh/irradiated vs fresh/non-conditioned p = 0.007; thawed/irradiated vs thawed/non-conditioned p = 0.018; and fresh/irradiated vs thawed/irradiated p = 0.0054; fresh/non-conditioned vs thawed/non-conditioned p = 0.0051; Gehan-Breslow-Wicoxon Test). Rx = irradiation conditioning. (B) Plot shows the bone marrow chimerism (percentage of human CD45^+^ cells of total CD45^+^ cells) after the transplantation of AML samples from bone marrow (BM), peripheral blood (PB), and leukapheresis products (LPH) 12–16 weeks before. LPH-MNCs showed a significant increase in the bone marrow overall chimerism compared to BM-MNCs.

To optimize the transfer of non-separated AML cells from human patients into mice, we aimed at determining the optimal transplantation settings. We compared the engraftment of freshly isolated or stored MNCs from AML patients in non-conditioned or irradiated NSG recipient mice (Figure 1). MNCs of 19 primary AML samples were isolated from blood (PB, n = 6), bone marrow (BM, n = 9) and leukapheresis products (LPH, n = 6) (Table 1) and transplanted into NSG mice. BM chimerism larger than 0.1% was considered as successful AML engraftment [11], and was observed in almost all analyzed mice from all treatment groups (thawed AML/non-conditioned recipients 95.7%, thawed AML/irradiated recipients 100%, fresh AML/non-conditioned recipients 76.3%, fresh AML/irradiated recipients 100%). Strikingly, only about half of NSG recipient mice survived the scheduled time period after transplantation (12–16 weeks). The parameters promoting premature death was irradiation conditioning of the recipient mice and the use of fresh versus thawed donor-cells (Figure 1A). Thus, survival of the recipient mice depends on the conditioning regimen and the processing of the donor material.

**Table 1:** Characteristics of samples from 19 AML patients.

									blast-gate	CD3+	CD34+	CD34+CD38-	fresh		thawed	
no.	FAB	genetics	molecular aberration	clinical cstatus	source	leuco (Gpt/l)	gender	age (years)	% of hCD45+		% of blast-gate		no-Rx	Rx	no-Rx	Rx
1	4	int.	NPM1	initial	PB	64.4	F	66	64.2	2.29	10.7	0	X			
2	2	int.		refractory	BM	4.5	F	59	39.6	26.8	7.99	0.158	X			
3	6	int.		initial	BM	2.01	M	74	49.7	6.97	4.16	0.0258	X			
4	1	int.	NPM1	initial	BM	3.17	F	69	94.1	4.03	0.136	0.0317	X		X	
5	5	high		initial	BM	9.24	M	22	85.4	8.93	93.9	0.405		X		
6	5	int.	NPM1, FLT3-ITD	initial	LPH	46.85	M	59	83.8	6.57	0.266	0.0133	X		X	
7	1	int.	NPM1	initial	LPH	240.24	F	67	88.8	6.08	0.763	0	X		X	X
8	1	int.	NPM1	initial	PB	155	F	42	95.6	1.81	0.19	0				X
9	1	int.	NPM1	initial	PB	104.88	F	52	94.3	2.19	7.79	0.136	X		X	X
				initial	BM				94.2	2.35	7.27	0.091				
10	6	int.		initial	BM	10.48	M	53	13.5	16.9	2.35	0.0417	X	X	X	X
11	1	int.	NPM1, FLT3-ITD, FLT3-TKD	initial	LPH	135.19	F	70	90.1	2	44.4	0.0942			X	X
				relapse	PB	59.3	F	70	92.7	2.66	36.8	2.22		X		
12	2	int.		initial	PB	44.08	M	72	93	0.293	82.4	0	X	X		
13	5	int.	NPM1, FLT3-ITD	initial	LPH	337.31	F	49	93.9	4.76	3.29	0.0234			X	
14	2	int.		refractory	BM	2.9	M	76	69.2	7.34	22.3	0.0343	X	X		
15	n.a.	int.		relapse	PB	36.69	M	69	91.4	1.48	61.9	0.486	X	X		
16	1	high		initial	BM	3.81	F	70	76.7	7.86	36	0.0236		X		
17	5	int.	NPM1	relapse	LPH	203.64	M	61	93.2	2.27	0.044	0.00161	X	X		
18	0	int.		initial	LPH	132.11	M	73	94.5	1.89	71.5	0,8181	X	X		
19	2	high		relapse	BM	1.61	F	51	28	45.2	93.7	19.6	X	X		

BM = bone marrow, F = female, FAB = French-American-British-classification, FLT3-ITD = Internal tandem duplication of FLT3, FLT3-TKD = Tyrosine kinase domain mutation of FLT3, Int. = intermediate, LPH = leukapheresis, M = male, n.a. = not available, NPM1 = nucleophosmin1 mutated, PB = peripheral blood, Rx = irradiation.

To assess for potential influence of graft-processing and transplantation procedure on the engraftment kinetics and quantity we compared engraftment of donor-cells between the four treatment groups. There were no changes concerning the maximum donor-cell chimerism, reconstitution kinetics in the blood of the recipient mice, and donor-cell chimerism in the bone marrow at the time point of analysis (i.e. at the planned sacrifice or the premature death of the animals)(data not shown). Source of donor-cells influenced chimerism in the bone marrow of the recipient mice in favor for leukapheresis (LPH)-derived donor cells over bone marrow donor cells (Figure 1B). However, maximum peripheral blood chimerism, reconstitution kinetic, overall survival of the recipient mice, and the type of engraftment were again not statistically different between the different graft sources (Figure S1A-D).

We conclude that the extent and kinetic of the engraftment of MNCs from human AML patients is independent of sample processing or conditioning of the recipient mice. However, sample processing and conditioning of the recipient mice are crucial factors determining survival of the recipient mice.

### Heterogeneity in Extent and Engrafted Cell types after Transplantation of Mononucleated Cells from AML Patients

**Figure 2:**
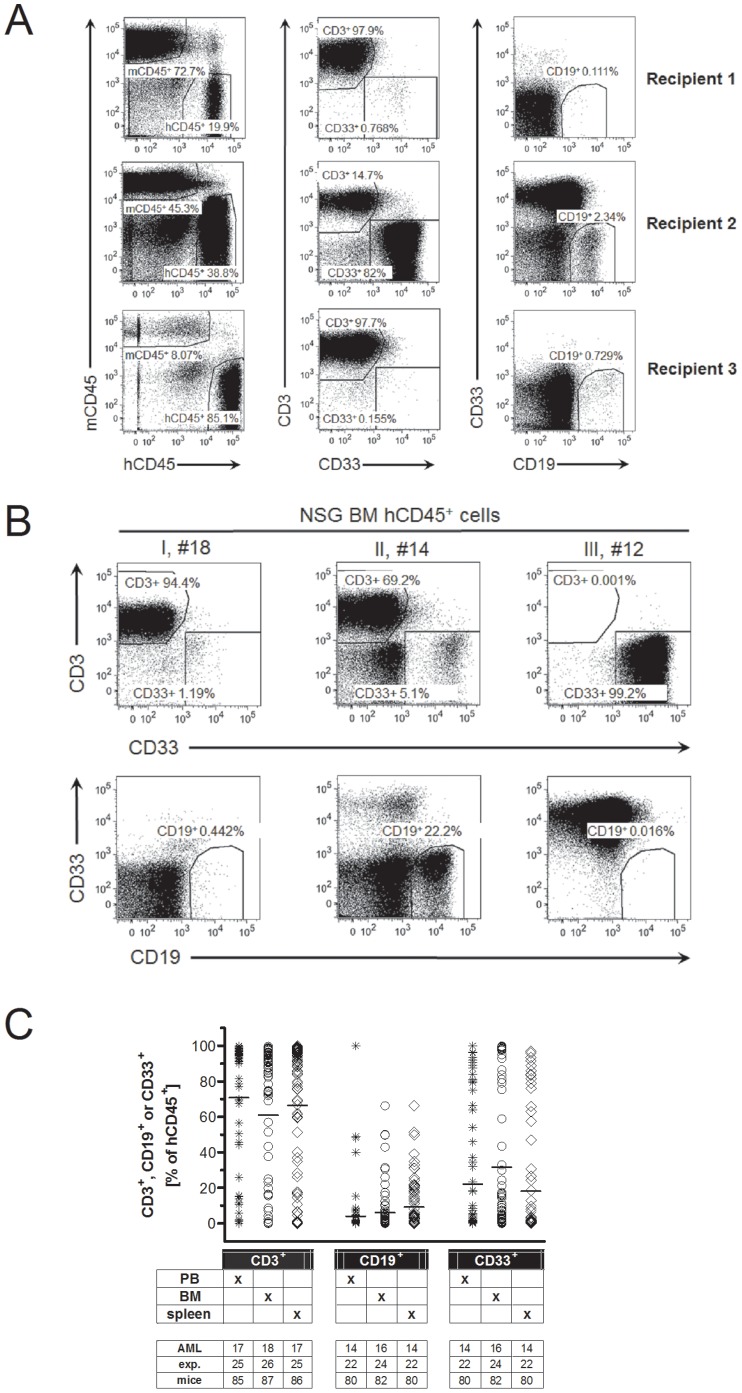
Intra- and inter-sample heterogeneity in the engraftment of MNCs from AML patients. (A) Dot plots show the frequency of human leukocytes in the bone marrow of three NSG recipient mice that were transplanted with MNCs from the blood from AML patient #9 (Table 1), 7–10 weeks before (left column). Human cells were further analyzed for the cell surface expression of CD3 versus CD33, and CD33 versus CD19 (middle and right column, respectively). (B) Dot plots show human leukocytes in the bone marrow of NSG recipient mice that were transplanted with MNCs from LPH, BM or PB from AML patients #18, #14, and #12 (Table 1), respectively. Human leukocytes were analyzed for the expression of CD3, CD19 and CD33 six weeks (AML #18 and #14) or ten weeks (AML #12) after transplantation. (C) Frequency of CD3, CD19 or CD33 positive human leukocytes in the blood (PB, asterix), bone marrow (BM, circles) and spleen (diamonds) of NSG mice are presented as percentage of human CD45^+^ cells. The number of AML-samples (AML), independent experiments (exp.), and recipient mice (mice) is indicated.

To analyze the reproducibility and the quality of engraftment we transplanted the same number of MNCs from AML patients into several NSG recipient mice and analyzed the frequency of human leukocytes. Results of a representative AML (AML #9, Table 1) transplantation is shown in Figure 2A. We found large differences in the magnitude of engraftment between recipients of the same donor-cells (20%, 39%, and 85%). Furthermore, analysis of the cell surface expression of CD3, CD19, and CD33 on human leukocytes in the bone marrow of recipient mice also revealed a striking difference between recipients of the same donor-cells that ranged from very low (<1%, Figure 2A, top) to very high (82% middle) frequencies of myeloid cells (CD33^+^). Consistently, CD3^+^ T cells and CD19^+^ B cells were also found to different frequencies.

Consequently, we observed a very high variability in the engraftment of different AML samples (Figure 2B). (I) Mainly CD3^+^ cells, (II) multilineage engraftment including CD3^+^, CD19^+^ and CD33^+^ cells, and (III) robust engraftment of CD33^+^ cells. However, in the bone marrow, blood and spleen of most of the recipient mice, we mainly detected CD3^+^ cells (Figure 2C). B-lymphocytes (CD19^+^) and myeloid cells (CD33^+^), which may include potential AML blasts represented a minority of human leukocytes in all analyzed organs. To test whether the cellular composition of the graft determines the type of engraftment, we analyzed in a large cohort of recipient mice whether the frequency of T cells, AML blasts in the graft, or total leukocytes in the patients correlated with dominant T cell or myeloid cell engraftment in the recipient mice. None of the tested graft parameters correlated significantly with the type of engraftment in the recipient mice (Figure S2), suggesting that a stochastic mechanism determines the type of engraftment. Taken together, engraftment of AML samples in NSG mice shows a large heterogeneity with respect to the extent and quality of the engraftment.

In conclusion, using 19 AML samples we showed that the extent of engraftment and the cellular composition of the engrafted human leukocytes are independent from the cellular composition of the graft, and that irrespective of the patient sample used, mainly human CD3^+^ cells are found in blood, bone marrow and spleen of recipient mice.

### CD3^+^ Cells are Donor-derived Polyclonal T Lymphocytes

**Figure 3:**
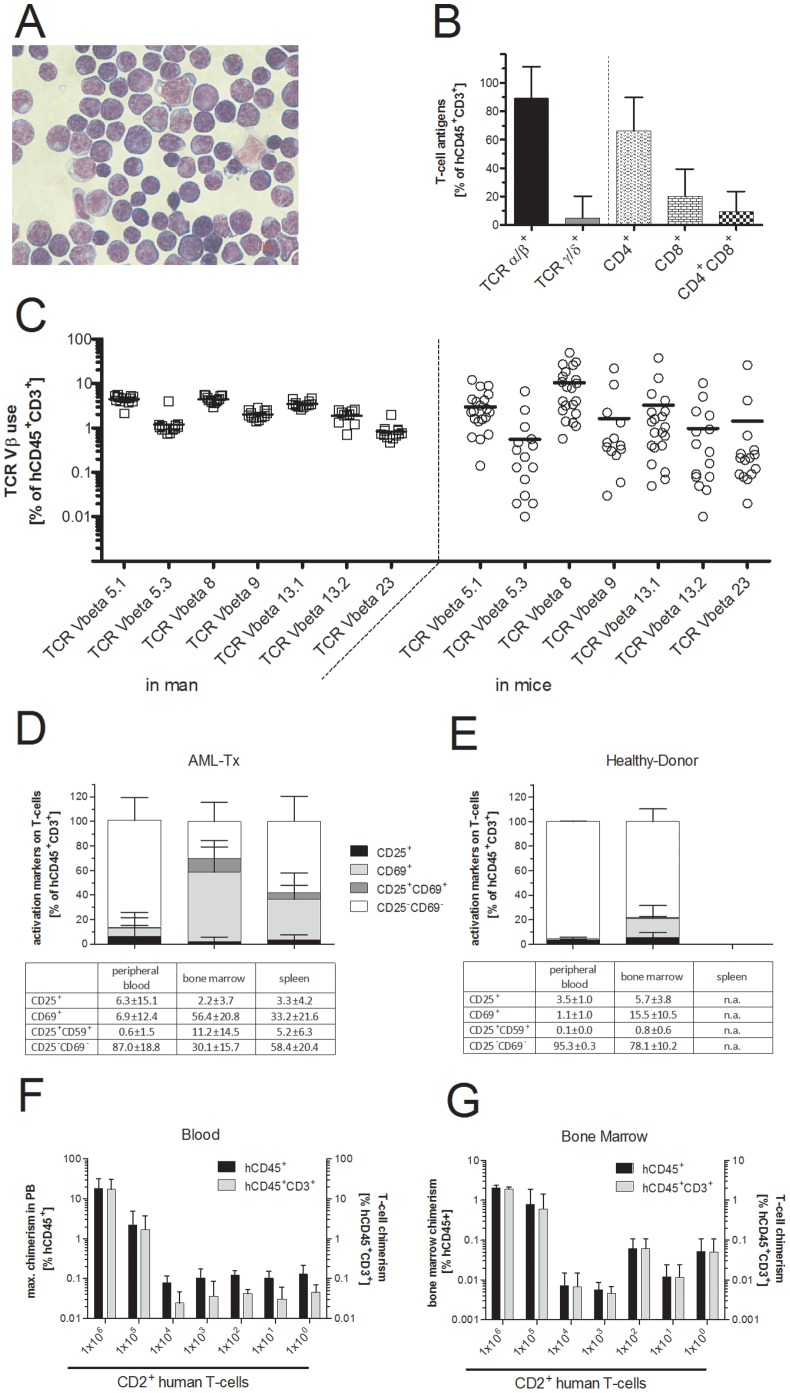
Human CD3^+^ cells in NSG recipient mice are polyclonal activated T lymphocytes. (A) Human CD3^+^ cells are morphologically diverse. Sorted human CD45^+^CD3^+^ cells (sort purity: 98.6%) isolated from the bone marrow of a NSG recipient mouse that had received 5×10^6^–10^7^ freshly isolated MNCs from AML #10 three weeks before were cytospun and stained according to May-Grünwald Giemsa. (Graft phenotype in the recipient: bone marrow: 35% hCD45^+^; composition of human leukocytes: CD3^+^ = 93%, CD19^+^ = 0.8%, CD33^+^ = 0.8%; surface antigens on CD3^+^ cells: CD4^+^ = 80%, CD8^+^ = 14%, CD4^+^/CD8^+^ = 3%, TCRα/β^+^ = 99%, TCRγ/δ^+^ = 0.02%). Photograph is representative for 6 different AML samples. Scale bar indicates a section of 10 micrometers. (B) Human CD3^+^ cells are identified as T lymphocytes by the surface expression of the T cell receptors (TCRs) and co-receptors. Graph shows a summary of the expression of TCRα/β or TCRγ/δ, and the expression of CD4 and CD8 on CD3^+^ cells the bone marrow of NSG mice that were transplanted with AML-MNCs as described in (A) (Within CD3^+^ cells: TCRγ/δ: 5.0±15.2%, TCRα/β: 89.2±22.1%; Within TCRα/β^+^ cells: CD4^+^ CD8^−^: 66.2±23.6%, CD4^−^ CD8^+^: 20.2±19.2%, CD4^+^ CD8^+^: 9.3±14.5%.) (C) Plot shows the frequency of indicated Vβ segments used in human α/β T cell receptors on T cells in the blood of healthy controls (‘in man’) and in the bone marrow of NSG mice that had received MNCs from AML patients as described in (A, ‘in mice’). Frequencies based on human CD45^+^ CD3^+^ cells are shown. (D) Bar graph shows the frequency of human T lymphocytes (hCD45^+^CD3^+^) that express CD25 and CD69 in blood, bone marrow and spleen of NSG mice that were transplanted with 10^7^ MNCs from AML patients 9.7±2.2 weeks before. N = 53 for all groups. (E) Bar graph shows the frequency of T lymphocytes that express CD25 and CD69 in the peripheral blood (4.7±0.3% of T lymphocytes) and bone marrow (21.9±10.2%) of healthy donors. N = 3 for all groups. (F) Maximum blood chimerism was determined after the transplantation of titrated numbers of CD2^+^ T cells that were sorted from the peripheral blood of a healthy donor. Donor-cell chimerism ≥0.1% hCD45^+^ (considered T cell engraftment, left y-axis) was reached only if ≥10^5^ CD2^+^ cells were transplanted. Virtually all engrafted human leukocytes expressed CD3 (% hCD45^+^CD3^+^, right y-axis). Mean and standard deviation are shown. (G) Plot depicts donor-cell chimerism in the bone marrow of NSG recipient mice as described in (D). Mean and standard deviation are shown.

To determine the identity of hCD45^+^CD3^+^ cells we performed a morphological analysis (Figure 3A), and found typical lymphoid cells (small cells, large nucleus, few cytoplasm, dark blue stained cytoplasm) as well as cells with a different morphology (large cells with large nuclei, more cytoplasm and lightly blue stained cytoplasm), suggesting that hCD45^+^CD3^+^ cells contain morphologically distinct populations, potentially including activated T lymphocytes. CD3 was not aberrantly expressed on AML blasts in the transplanted material (not shown), suggesting that hCD45^+^CD3^+^ cells are not diseased cells but rather expanded, graft-derived co-transplanted T cells. Consistent with this interpretation, CD3^+^ donor cells could be separated into subpopulations expressing either the alpha/beta T cell receptor (TCRα/β) or the gamma/delta TCR (TCRγ/δ). Furthermore, TCRα/β^+^ cells were a heterogeneous population that included CD4 or CD8 single positive or CD4 CD8 double positive cells (Figure 3B), confirming the presence of distinct T lymphocyte populations in the recipient mice.

Next, we asked whether human T cells in NSG recipient mice were of mono- or oligoclonal origin. Most T cell malignancies are characterized by a monoclonal expansion of T lymphocytes [26], whereas the simultaneous activation of T cells that occurs for example in the context of graft versus host disease (GvHD) usually represents an at least oligoclonal event [27]. To discriminate between T cell neoplasm and non-malignant causes for the increase of T cell numbers after transplantation, we analyzed the use of variable segments of the TCR beta chain (Vβ) on CD3^+^ T cells in the blood of healthy individuals and NSG mice that were transplanted with MNCs from AML patients (Figure 3C). In man, TCR Vβ chain usage was very uniform between individuals, and the mean prevalence of the TCR Vβ chain in mice was similar to the distribution observed in healthy donors, suggesting that an at least oligoclonal expansion of co-transplanted T cells is the basis for the over-representation of CD3^+^ T cells in the human graft.

We conclude that engrafted hCD45^+^CD3^+^ cells are polyclonal T cells that show the same relative distribution of Vβ chain usage compared to man, suggesting that mature co-transplanted T cells expanded in the recipient mice.

### Donor-derived Co-transplanted T Cells Induce Xenogeneic Graft Versus Host Disease

To determine whether the symptoms of prematurely diseased mice depended on diseased myeloid cells or expanded T lymphocytes, we analyzed leukocyte engraftment in unconditioned mice that had received MNCs from healthy donors (Figure S3). Irrespective of the graft source (PB or BM or PBMC), 82% of recipients of MNCs from healthy patients developed the same symptoms, as did mice that were transplanted with MNCs from AML patients, and showed a reduced survival accompanied by pale bones harboring reduced numbers of cells, and enlarged spleens (Figure S3 and S4). However, NSG mice that had received MNCs from healthy donors showed differences in the overall human chimerism (hCD45^+^) in the analyzed organs (Figure S4B), but virtually all engrafted human leukocytes where CD3^+^ T lymphocytes (>93% in PB, BM and spleen, Figure S4C). Further, the majority of mice that deceased prematurely tended to have a lower myeloid chimerism (27.9%±23.9% vs. 40.0%±24.3%) and a higher T cell chimerism (58.6%±23.7% vs. 1.5%±3.0%) compared to mice that survived until the scheduled end of the experiment without signs of illness. Therefore, an elevated T cell chimerism seems to be predictive for premature death, and these findings support the interpretation that disease symptoms are induced by expanded co-transplanted donor-derived T lymphocytes.

Human T lymphocytes in NSG mice that received MNCs from healthy donors showed a similar expression of the co-receptors and diversity in the TCR Vβ segment use compared to T cells in NSG recipients of AML-MNCs (Figure S4D and E), suggesting that after the transplantation of MNCs from healthy individuals into NSG recipient mice polyclonal human T lymphocytes expanded.

The symptoms of prematurely deceased mice are similar to those suffering from xGvHD [28]–[34]. Because the activation status of donor T lymphocytes directed against host antigens is a central part of the pathophysiology of GvHD [35] and xGvHD [36], we further analyzed for surface activation-markers CD25 and CD69 in the blood, BM and spleen of recipient mice (Figure 3D,E) [35], [36]. In the blood only a small fraction of hCD45^+^CD3^+^ cells expressed activation markers, whereas, in the bone marrow and spleen large fractions of donor-derived T cells were activated after the transplantation of MNCs from AML or healthy donor-derived cells (Figure 3D and Figure S4F). Organ-specific imbalance in the frequency of activated T cells was observed to a much lower extent in healthy donors (Figure 3E), suggesting that T cells from both donor groups were activated after transfer, and that the pathophysiology of xGvHD in all NSG recipient mice had the same cause.

Bone marrow hypoplasia, a dominant sign of xGvHD [31], occurred more pronounced in NSG recipient mice that had received MNCs from healthy donors compared to AML donors (Figure S3). To assess whether this effect was mediated by different T cell doses that were co-transplanted with MNCs from AML patients (7.5±8.4×10^5^, n = 14) or healthy donors (27.4±23.5×10^5^, n = 9), we transplanted titrated numbers of sorter-purified human CD2^+^ T cells into NSG recipient mice and analyzed human T lymphocyte frequencies in the blood (Figure 3F) and bone marrow (Figure 3G) of the recipient mice. Human T cell engraftment (≥0.1% CD3^+^ T cells within the fraction of human leukocytes) was exclusively detected in mice that were transplanted with ≥10^5^ T cells. Maximum chimerism of the peripheral blood and BM chimerism correlated with the number of transplanted CD2^+^ cells (Figure 3F and E). However, other factors seem to influence T cell engraftment since there was a lack of correlation between the number of engrafted T cells and co-transplanted T lymphocytes after the transfer of MNCs from AML patients (Figure S2A).

We conclude that co-transplanted donor T lymphocytes expanded in NSG recipient mice, became activated and mediated xGvHD that occurred independent from AML disease in the donor.

### Engrafted T Lymphocytes are not of Acute Myeloid Leukemia Origin

Malignant transformation of hematopoietic precursors is considered as the underlying cause of leukemogenesis [37]–[40]. To discriminate between regular and disease-related hematopoiesis and to determine whether mutant *Npm1*, a founder genetic lesion in AML, ultimately leads to the proliferation of diseased cells of the myeloid and lymphoid lineages [41], [42], we transplanted MNCs from three patients that carried mutant *Npm1* into mice and monitored for the molecular aberration in cells of the myeloid and lymphoid lineages at the time point of analysis.

Three AML samples with *Npm1* mutations (#7–9, Table 1), which had shown mixed engraftment in previous experiments, and from which sufficient primary material was still available were chosen to elucidate the origin of engrafted human myeloid and lymphoid cell types (CD33^+^, CD3^+^ and CD19^+^). AML-MNCs were transplanted in sublethally irradiated mice. Molecular analysis from sorted donor-derived cells from the bone marrow of recipient mice revealed that only donor-derived myeloid cells, but not T or B lymphocytes carried mutant *Npm1* alleles, suggesting a distinct cellular origin of cells of the myeloid and lymphoid lineages at the time point of differentiation (Table 2). These results confirm the independence of T cell expansion from AML diseased cells, and suggest that the putative AML-initiating driver mutation occurred within a cell that could not give rise to lymphocytes any more, thus the cells of origin of these leukemias were not multipotent, but myeloid-restricted hematopoietic precursor cells [41].

**Table 2:** Mutational analysis of *Npm1* in engrafted human leucocytes.

	CD3^+^ T cells	CD19^+^ B cells	CD33^+^ myeloid cells
**AML #7**	Wild type	Wild type	mutant
	Wild type	Wild type	mutant
**AML #8**	Wild type	Wild type	mutant
	Wild type	Wild type	mutant
	Not available	Wild type	Not available
**AML #9**	Wild type	Wild type	mutant
	Wild type	Wild type	mutant

Thawed MNCs of *Npm1*-mutated AMLs #7–9 were transplanted into irradiated NSG recipients. Engrafted CD19+ B- and CD3+ T lymphoid and CD3- CD19- CD33+ myeloid cells were sorted (sort purity >99% for each fraction) and analyzed for presence of mutant *Npm1*. T- and B-lymphocytes in the bone marrow of the recipients displayed wild-type *Npm1*, while myeloid cells were positive for mutated *Npm1*.

### Depleting CD3^+^ T Cells from the Graft Strengthens AML Engraftment

**Figure 4:**
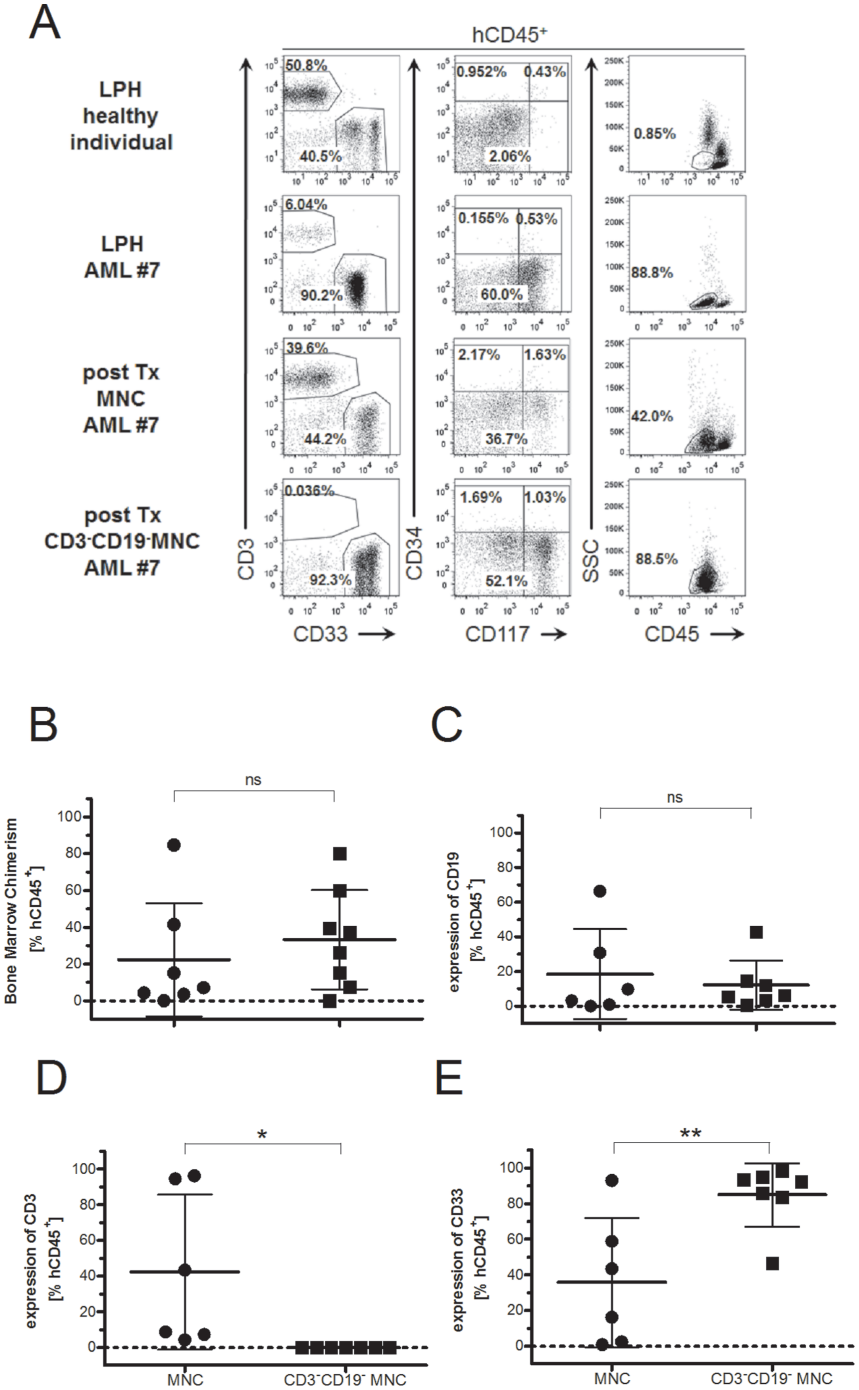
Depletion of CD3^+^ and CD19^+^ cells from the graft prevents xGvHD symptoms, and augments AML engraftment. Unconditioned NSG mice were transplanted with equal numbers (0.15–0.6×10^7^ cells) of thawed MNCs from AML #7–9 (Table 1) with or without previous depletion of CD3^+^ and CD19^+^ lymphocytes (CD3^−^CD19^−^MNC and MNC, respectively). Mice were sacrificed for bone marrow analysis 12 weeks after transplantation, or when detoriation of health occurred, and analyzed by flow cytometry. AML samples #7–9 showed a common, characteristic phenotype of *Npm1*-mutated AML with positivity for CD33 and CD117, but lack of CD34 expression. (A) Dot plots show expression of CD3 versus CD33 (left column), CD34 versus CD117 (middle), and side scatter (SSC) versus CD45 (right column) on human CD45^+^ MNCs from a healthy donor (top), from AML patient #7 (second row from top), and from the bone marrow of NSG recipient mice that were transplanted with either MNCs (second row from bottom) or CD3^−^CD19^−^MNCs (bottom) from AML #7 twelve weeks before. Data representative for all AML samples #7–9 analyzed. (B-E) Plots show the mean donor-cell chimerism (B, % hCD45^+^; MNC: 33.3±27%, CD3^−^CD19^−^MNC: 22.4±30.9%), the frequency of CD19^+^ cells (C, MNC: 18.5±26.0%, CD3^−^CD19^−^MNCs: 12.3±14.3%), or CD3^+^ cells (D, MNC: 42.4±43.5%, CD3^−^CD19^−^MNCs: 0.02±0.02%, p = 0.0247), or CD33^+^ cells (E, MNC: 35.9±36.3%, CD3^−^CD19^−^MNCs: 85.0±17.8%, p = 0.0087) within donor-derived leukocytes (hCD45^+^) in the bone marrow of recipient mice 6–12 weeks after transplantation with AML-samples #7–9 (MNC: 33.3±27%, CD3^−^CD19^−^MNC: 22.4±30.9%).

We showed that engraftment of diseased myeloid cells can occur independently from the engraftment of lymphoid cells in NSG mice (Table 2). To determine whether xGvHD can be circumvented and whether we can augment the engraftment of AML-diseased cells, we transplanted MNCs from AML patients #7–9 that where sorter-depleted for T and B lymphocytes (sort purity >99.5%) in sublethally irradiated recipients, and analyzed lymphoid and myeloid engraftment in the recipient mice 12 weeks later (Figure 4). AML blasts of patient #7–9 shared a common, characteristic phenotype of *Npm1*-mutated AML: CD33^+^CD34^−^CD117^+^. This phenotype was distinguishable from the surface profile of MNCs from a healthy individual, and was therefore used as a marker to follow diseased AML cells after transplantation (Figure 4A). At the time point of analysis, co-transplanted T lymphocytes had expanded and constituted a large proportion of the graft, whereas no T cells were detected in the bone marrow of recipient mice that had received lymphocyte-depleted MNCs from AML #7–9. Moreover, in recipients of CD3^−^CD19^−^ MNCs >90% of all engrafted human leukocytes were of the myeloid lineage (CD33^+^) and the majority of engrafted human myeloid cells showed the same phenotype compared to diseased AML #7–9 before transplantation, suggesting that predominantly diseased AML cells engrafted and expanded. Consistent with this interpretation a very high percentage of human leukocytes localized in the ‘blast gate’ (SSC versus CD45), that is clinically used for the identification of diseased leukemic cells, in recipient mice of lymphocyte-depleted MNCs but not in recipients of MNCs. There were no differences in bone marrow chimerism (Figure 4B) or the percentage of CD19^+^ cells in the recipient mice (Figure 4C). In contrast, CD3^+^ cells significantly decreased after the transfer of lymphocyte-depleted MNCs in all experiments (Figure 4D), preventing the development of xGvHD. Consequently, none of the mice receiving CD3^−^CD19^−^ MNCs showed deterioration of health, while two mice transplanted with non-fractionated AML-MNCs had to be sacrificed ahead the scheduled end of the experiment (5.8 and 6.4 weeks after transplantation, respectively). Furthermore, the engraftment of CD33^+^ cells was significantly increased after the transplantation of lymphocyte-depleted MNCs compared to non-depleted MNCs (Figure 4E), suggesting that the engraftment of diseased myeloid cell types was augmented in all experiments. Again, mice transplanted with non-fractionated AML-MNCs tended to have a larger spleen compared to mice transplanted with CD3/CD19-depleted MNCs (murine *and* human leukocytes (CD45^+^): 0.68±1.09×10^6^ cells vs. 0.22±0.17×10^6^ cells; not statistically significant). The bone marrow cellularity of mice transplanted with CD3/CD19-depleted cells was non-significantly reduced compared to control animals (murine *and* human leukocytes (CD45^+^): 1.35±0.34×10^6^ cells vs. 1.65±0.17×10^6^ cells), whereas mice receiving non-fractionated AML-MNCs showed a significant reduction in cellularity (1.2±0.24 x10^6^ cells).

In conclusion, depleting T cells from AML grafts, prevents the expansion of T cells and the occurrence of xGvHD in the recipient mice and leads to a uniform engraftment of diseased CD33^+^ myeloid cells.

## Discussion

A robust assay to test for human leukemia re-initiating cells *in vivo* is lacking. Despite the differences between mice and men the use of mice as recipients for leukemia samples will allow the analysis of human tumor biology in an *in vivo* microenvironment. In contrast to previous publications [43]–[45], we show here using NSG recipient mice, that a robust engraftment of human myeloid cell types is a rare event upon the transplantation of MNCs from AML patients and that the same donor sample can show different patterns of engraftment in distinct recipient mice. We show that co-transplanted, polyclonal T lymphocytes expand and we further define a threshold for the transfer of donor T cells that is compatible with the transplantation of AML cells. Last, we show that engraftment of myeloid cells can be obtained through the depletion of T lymphocytes from the graft, thereby transferring all other cells that may have the potential to re-initiate a human tumor in mice.

It has become evident over the last few years that a common cell surface phenotype identifying cancer stem cells in AML is missing [7]–[11], suggesting that either the cancer stem cell theory is not applicable or that the correct phenotype still awaits identification. Furthermore, coating of human cells with antibodies might also influence the engraftment capacity of stem cells [10]. In conclusion, lack of a common cell surface phenotype for cancer stem cells also means that the transplantation of titrated numbers of bulk tumor cells into mice is necessary to obtain information on the frequency of leukemia initiating activity in AML samples. Further, recent data suggest that the use of suboptimal recipient mice lead to an underestimation of cancer stem cell frequencies, emphasizing the necessity to re-assess the presence, function and frequency of this activity in AML samples [7]–[11]. Therefore we aimed at re-addressing engraftment of human cells after the transplantation of MNCs from AML patients into NSG recipient mice. We frequently detected engrafted human cells in the bone marrow, blood and spleen of recipient mice as reported by other laboratories [43]–[49]. However, in contrast to previous reports [45], [46], [49], the capacity of human cells to engraft in mice failed to correlate with the graft source, AML subtype, transplantation procedure, or sample processing. Using MNCs as donor material we found a striking variability in terms of engrafted donor-derived cell types (T or B lymphocytes or myeloid cells) in recipient mice. Irrespective of the cause for heterogeneity of engrafted cells, this finding suggests that MNCs from AML patients are inappropriate graft cells for the study of cancer stem cell frequency and biology.

For the study of a human myeloid cell type-based disease in mice, human myeloid cells need to engraft. However, in contrast to previous studies [43]–[45], we report here that the transplantation of MNCs from AML patients into NSG mice led to a predominant engraftment of human T cells. Myeloid cells, including putative diseased cells, only represented a minority of engrafted human leukocytes. It has been suggested that tumor re-initiating activity of AML samples resides in monoclonal T lymphocytic leukemia re-initiating stem cells that expand in NSG recipient mice [50]. However, we showed that the donor-derived T cells were at least polyclonal and lacked expression of the molecular marker of the transplanted AML cells. Others reported on the appearance of only a minor population of T cells in a small fraction of recipient mice after the transplantation of MNCs from AML patients [45]. This work was restricted to AML samples with >80% blasts, which might contribute to the differences in lymphoid and myeloid engraftment compared to our results. However, in our cohort even samples with >80% blasts showed expansion of human CD3+ cells. Further studies focused on the detection of human leukocytes (hCD45+ [43], [46]) or myeloid but not lymphoid cells of human origin [44] as a measure for human donor cell engraftment. Thus, the frequency of lymphoid donor-derived cells in those studies is not known.

Taken together, we show here that the transplantation of MNCs from AML patients frequently results in the polyclonal expansion of co-transplanted T lymphocytes but not in engraftment of cells of the myeloid lineage.

Expansion of human T cells in mice has been reported in studies focusing on the establishment of xGvHD models by transplanting MNCs of healthy donors [28]–[31], [34], [36], [51], [52]. Comparing two mouse strains, Ali and coworkers were able to demonstrate that NSG mice are especially susceptible to xGvHD [51]. Interestingly, the incidence of xGvHD seems to be associated with the HLA class-I alleles expressed by the human donor [52]. Similar to our data, the predominant expansion of human T cells prevented the engraftment of non-malignant hematopoietic stem cells [32]. Nevertheless, predominant T cell engraftment after transplantation of MNCs from patients with malignant myeloid diseases was apparently also observed by others, as T cell depletion is described in the ‘material and method’ section of some studies [53]–[56]. Furthermore, lethal xGvHD was also observed when transplanting human MNCs to achieve engraftment of chronic lymphocytic leukemia (CLL) suggesting a similar phenomenon for lymphoid diseases [57]. Nevertheless, T cell expansion after transplantation of AML-MNCs or MNCs from patients with low-risk myelodysplastic syndrome (MDS) has only been incompletely described so far [54]–[56]. Consistent with our data the foremost phenotype of xGvHD in NSG mice is bone marrow hypoplasia accompanied by peripheral cytopenia most likely causing death [31], [51], [52]. We showed that donor-derived T lymphocytes from healthy donors and from AML patients acquire an activated phenotype in mice, suggesting that xenoreactivity may cause expansion by activation. Such a memory-type T lymphocyte phenotype is found in models of xGvHD [51]. Even though higher numbers of transplanted T cells were reported to aggravate xGvHD [31] there was no evidence for a correlation between T cell chimerism and transplanted donor T cell numbers, which may be explained by a generally lower number of T cells that were co-transplanted with the MNCs from AML patients, compared to the previously reported minimum dose of T cell inoculum necessary for engraftment [31]. Nevertheless, and in agreement with King et al. we also observed a significant shortened median survival time of sublethally irradiated recipients of AML-MNCs [31], supporting the conclusion that xenoreactive donor-derived T cells expanded polyclonally and led to death of the recipient mice.

In conclusion, we showed that the transfer of MNCs from AML patients is not suitable for the analysis of frequency and biology of tumor re-initiation activity in mice. Relative to previous attempts to improve the engraftment of diseased human cells of the myeloid lineage [54], we detected a significant increase in engrafted diseased myeloid cells, suggesting that the transplantation of T cell depleted MNCs from AML patients into NSG mice may represent a robust model to re-assess presence, frequency and function of leukemia stem cells.

## Materials and Methods

### Human Samples: Ethics Statement

Samples from healthy volunteers and AML patients were obtained from patients at the University Hospital Dresden with written informed consent according to protocols approved by the Institutional Review Board (IRB), the Ethikkommission an der TU Dresden. MNCs of peripheral blood (PB) and bone marrow (BM) were isolated by density centrifugation on a Ficoll gradient. Residual red blood cells of peripheral blood mononuclear cells (PBMC) and leukapheresis products (LPH) were lysed (ACK buffer, Invitrogen). Nucleophosmin (*Npm1*) and FMS-like tyrosine kinase 3 (*Flt3*) mutation analysis was performed as described before [21], [22].

### Mice

NOD.Cg-*Prkdc^scid^ Il2rg^tm1Wjl^*/SzJ (NOD/SCID/IL2rγ^−/−^, NSG) mice were purchased from The Jackson Laboratory (Jackson Laboratory, Bar Harbor, Maine, USA). NSG mice are characterized by the absence of mature T-, B- and NK-cells and show additional defects in innate immunity [3], [4]. Mice were kept under specific pathogen-free (SPF) conditions in sterilized microventilated cages with sterilized food and water ad libidum in the animal facility at the Medical Theoretical Center of the University of Technology Dresden. Experiments were performed in accordance with German animal welfare legislation, and were approved by the relevant authorities, the Landesdirektion Dresden (24-9168.11-1/2009-33). Cell suspensions were prepared as described previously [23]. Briefly, spleen and thymus were disintegrated by gentle disruption between glass slides, while femuras and tibias were crushed using a mortar. Red blood cells from spleen and bone marrow samples were removed by lysis. May-Grünwald Giemsa staining was performed as described before [23].

### Flow Cytometry

Samples were prepared as described before [23] using the following antibodies recognizing human antigens (clones given in brackets): CD45 (HI30, BioLegend), CD2 (RPA-2.10), CD19 (HIB19), CD34 (581), CD45 (HI30), TCRαβ (T10B9.1A-31, all BD Biosciences), CD3 (UCHT1), CD3 (SK7), CD4 (SK3), CD8a (RPA-T8 eB), CD25 (BC96), CD33 (HIM3-4), CD38 (HIT2), TCRγδ (B1.1 eB), TCR Vβ 5.1 (LC4), TCR Vβ 5.3 (3D11), TCR Vβ 8 (JR-2), TCR Vβ 9 (AMKB1-2), TCR Vβ 13.1 (H131), TCR Vβ 13.2 (H132) and TCR Vβ 23 (AF23), anti-mouse CD45 (30-F11, all eBioscience). FITC conjugated streptavidin was purchased from BD Biosciences. Doublet discrimination was routinely carried out and dead cells were excluded by 4, 6 diamidino-2-phenylindole (DAPI)-staining. Analysis or sorting was performed on BD LSRII or AriaII machines, respectively (BD Biosciences). Purity of the CD3^−^CD19^−^ population was 100% (AML #7), 99.6% (AML #8) and 100% (AML #9). For T cell limiting dilution transplantations, indicated numbers of CD2^+^ T lymphocytes (98.2% purity) from PBMC of healthy donors were transplanted. Cells for molecular analysis of *Npm1* were sorted to 98%±2% (CD3^+^, n = 6), 98.8%±0.8% (CD19^+^, n = 7), and 92%±9% (CD33^+^, n = 7) purity. At least 300 cells were used for mutation analysis of *Npm1* as described before [24].

### Transplantation

The age of mice used for experiments ranged between 3 to 6 weeks. Cell suspensions containing 5×10^6^–10^7^ MNCs in 200 µl PBS/5% FCS were injected intravenously as described before [23]. 0.15–0.6×10^7^ CD3^−^CD19^−^ AML-MNCs were transplanted in indicated experiments. For the first 3 weeks after transplantation, water was supplemented with neomycin (1.17g/l, Sigma). In three experimental series mice were transplanted without prior conditioning. To analyze factors that potentially influence engraftment, we compared the engraftment kinetics and the phenotype of human cells in non-conditioned and irradiated recipient mice after transplantation of AML-MNCs (Figure 1A). Furthermore, cells from healthy donors were exclusively transplanted in unconditioned mice to avoid excess of xenogeic Graft versus Host Disease (GvHD) induced death (1. influence of the transplanted T cell dose on the occurrence of xenogeneic GvHD, Figure 3 F-G; 2. engraftment kinetics and phenotype of human cells after transplantation of MNCs of healthy donors, Figure S4 A-F). In all other experiments mice received 100cGy whole body irradiation (WBI), using an Yxlon MaxiShot X-ray irradiator (Yxlon), between 4 to 24 hours prior to human cell injection. Sublethal irradiation was chosen to enhance engraftment without the necessity of complete hematopoietic replacement [25]. As malignant cells have lost the ability to differentiate and to replenish all kind of fully differentiated functional blood cells, lethal irradiation would obligatory lead to death due to bone marrow insufficiency, irrespective of the engraftment of human AML. Mice were sacrificed 12–16 weeks after transplantation or earlier when detoriation of health occurred (decreased activity, poor grooming, weight loss, fur ruffling). All animal experiments were approved by the local relevant authorities (‘Landesdirektion Dresden’, Germany).

## Supporting Information

Figure S1 Source of AML-MNCs influences the engraftment kinetics of human leukocytes. MNCs from patients with AML were isolated from bone marrow (BM), peripheral blood (PB) or leukapheresis products (LPH), and 5×10^6^–10^7^ MNCs were transplanted freshly or after DMSO-based storage into non-conditioned or irradiated NSG mice. Recipient mice were sacrificed 12–16 weeks after transplantation or earlier when detoriation of health occurred. (A) The maximum human leukocyte chimerism in the blood during the observation period is presented in dependency of the graft source. (B) The graph shows the time point of the maximum peripheral blood chimerism in mice that received MNCs from bone marrow, blood or LPH samples from AML patients with AML. (C) Survival of mice transplanted with MNCs from bone marrow, blood or LPH samples from AML patients. (D) Plot depicts the frequency of human myeloid cells (CD33^+^) within all engrafted human leukocytes (hCD45^+^) according to the sample source.
(PDF)

Figure S2 Lack of correlation between the type of engraftment and the cellular composition of the graft. Frequencies of hCD45^+^ CD3^+^ (A) or hCD45^+^ CD33^+^ (B, C) positive cells in the bone marrow of NSG mice that were transplanted with 5×10^6^–10^7^ freshly isolated MNCs from 13 (A) or 11 (B+C) AML patients before. Frequency of CD3^+^ donor cells is depicted as a function of CD3^+^ cells in the graft (A). Frequency of CD33^+^ donor cells is depicted as a function of the number of AML blasts in the graft (side scatter^low^ CD45^+^, B) or total leukocytes of the patient (C).
(PDF)

Figure S3 Xenogeneic graft versus host disease leads to growth retardation, splenomegaly and bone marrow hypoplasia. (A) Picture shows NSG recipient mice that were transplanted 10^7^ MNCs (PB) from a healthy donor 6 weeks before (Tx) and non-transplanted control mice (ctrl.). Human donor-cell chimerism in the peripheral blood, bone marrow and spleen was 32%, 19% and 86%, respectively, and in all organs >98% of all human leukocytes expressed the CD3 antigen. Picture is representative for 5 independent recipient mice. (B) Femuras and tibias of mice that had received MNCs from healthy individuals as described in (A) were pale compared to control NSG mouse bones. (C) The spleen of mice that had received MNCs from healthy individuals was enlarged compared to a control spleen from a non-injected NSG mouse. Spleens depicted originate from mice described in (A). (D) Plot shows reduced bone marrow cellularity in mice that were transplanted with 5×10^6^–10^7^ MNCs from patients (left) or healthy donors (right). Bone marrow hypoplasia is detected in mice transplanted with MNCs from healthy individuals (1.8±1.4×10^7^) and from AML patients (2.8±2.0×10^7^), compared to non-transplanted NSG mice (middle, 4.3±1.8×10^7^). (E) Mean spleen cellularity of mice transplanted with MNCs of healthy donors (right, 4.6±9.1×10^7^) or AML patients (left, 4.9±8.4×10^7^) or untreated NSG mice (middle, 0.7±2.7×10^7^). Mice received grafts described in (D).
(PDF)

Figure S4 Transplantation of MNCs from healthy donors results in almost exclusive engraftment of human T lymphocytes. Unconditioned NSG mice were transplanted with 5×10^6^–10^7^ MNCs freshly isolated from the bone marrow and blood of healthy volunteers and leukapheresis products from G-CSF treated donors. Mice were sacrificed 12 weeks after transplantation or when status of health detoriated. (A) Mice transplanted with MNCs from healthy donors showed a significant shortened survival compared to mice transplanted with AML-MNCs. (B) Transplantation of MNCs of healthy donors led to organ specific chimerism that was determined at the time point of analysis. (C) Donor-derived leukocytes in the blood, BM and spleen of NSG mice that had received MNCs from healthy donors expressed predominantly CD3^+^ T lymphocytes, while CD19^+^ B-lymphocytes and CD33^+^ myeloid cells were barely detectable. (D) Plot shows the frequency of TCRα/β^+^ and TCRγ/δ^+^ T cells in CD3^+^ cells, and the frequency of the expression of CD4 and CD8 or both on TCRα/β^+^ T cells on donor-derived lymphocytes in the bone marrow of NSG recipient mice after the transplantation of MNCs from healthy donors. (CD4∶52.2±15.7%, CD8∶34.9±14.1%, CD4/CD8∶10.5±5.5%, TCRα/β: 97.3±9.5% and TCRγ/δ: 0.09±0.2%) (E) Plot shows the frequency of indicated Vβ segments in human α/β T cell receptors on T cells in the bone marrow of NSG mice that had received MNCs from healthy volunteers. Frequencies based on human CD45^+^ CD3^+^ cells are shown. (F) Bar graph shows the frequency of human T lymphocytes (hCD45^+^CD3^+^) that express CD25 and/or CD69 in blood, bone marrow and spleen of NSG recipient mice that were transplanted with MNCs from healthy patients. N = 26 for all groups.
(PDF)

## References

[1] Bosma GC, Custer RP, Bosma MJ (1983) A Severe Combined Immunodeficiency Mutation in the Mouse. Nature 301: 527–530. 6823332 10.1038/301527a0

[2] Feuring-Buske M, Gerhard B, Cashman J, Humphries RK, Eaves CJ, et al (2003) Improved engraftment of human acute myeloid leukemia progenitor cells in beta 2-microglobulin-deficient NOD/SCID mice and in NOD/SCID mice transgenic for human growth factors. Leukemia 17: 760–763. 12682634 10.1038/sj.leu.2402882

[3] Ishikawa F, Yasukawa M, Lyons B, Yoshida S, Miyamoto T, et al (2005) Development of functional human blood and immune systems in NOD/SCID/IL2 receptor {gamma} chain(null) mice. Blood 106: 1565–1573. 15920010 10.1182/blood-2005-02-0516 PMC1895228

[4] Shultz LD, Schweitzer PA, Christianson SW, Gott B, Schweitzer IB, et al (1995) Multiple Defects in Innate and Adaptive Immunological Function in Nod/Ltsz-Scid Mice. Journal of Immunology 154: 180–191. 7995938

[5] Wunderlich M, Chou FS, Link KA, Mizukawa B, Perry RL, et al (2010) AML xenograft efficiency is significantly improved in NOD/SCID-IL2RG mice constitutively expressing human SCF, GM-CSF and IL-3. Leukemia 24: 1785–1788. 20686503 10.1038/leu.2010.158 PMC5439963

[6] Dick JE (2008) Stem cell concepts renew cancer research. Blood 112: 4793–4807. 19064739 10.1182/blood-2008-08-077941

[7] Goardon N, Marchi E, Atzberger A, Quek L, Schuh A, et al (2011) Coexistence of LMPP-like and GMP-like leukemia stem cells in acute myeloid leukemia. Cancer Cell 19: 138–152. 21251617 10.1016/j.ccr.2010.12.012

[8] Martelli MP, Pettirossi V, Thiede C, Bonifacio E, Mezzasoma F, et al (2010) CD34+ cells from AML with mutated NPM1 harbor cytoplasmic mutated nucleophosmin and generate leukemia in immunocompromised mice. Blood 116: 3907–3922. 20634376 10.1182/blood-2009-08-238899

[9] Sarry JE, Murphy K, Perry R, Sanchez PV, Secreto A, et al (2011) Human acute myelogenous leukemia stem cells are rare and heterogeneous when assayed in NOD/SCID/IL2Rgammac-deficient mice. J Clin Invest 121: 384–395. 21157036 10.1172/JCI41495 PMC3007135

[10] Taussig DC, Miraki-Moud F, Anjos-Afonso F, Pearce DJ, Allen K, et al (2008) Anti-CD38 antibody-mediated clearance of human repopulating cells masks the heterogeneity of leukemia-initiating cells. Blood 112: 568–575. 18523148 10.1182/blood-2007-10-118331

[11] Taussig DC, Vargaftig J, Miraki-Moud F, Griessinger E, Sharrock K, et al (2010) Leukemia-initiating cells from some acute myeloid leukemia patients with mutated nucleophosmin reside in the CD34(-) fraction. Blood 115: 1976–1984. 20053758 10.1182/blood-2009-02-206565 PMC2837317

[12] Estey E, Dohner H (2006) Acute myeloid leukaemia. Lancet 368: 1894–1907. 17126723 10.1016/S0140-6736(06)69780-8

[13] Saito Y, Uchida N, Tanaka S, Suzuki N, Tomizawa-Murasawa M, et al (2010) Induction of cell cycle entry eliminates human leukemia stem cells in a mouse model of AML. Nat Biotechnol 28: 275–280. 20160717 10.1038/nbt.1607 PMC3857633

[14] Blair A, Hogge DE, Ailles LE, Lansdorp PM, Sutherland HJ (1997) Lack of expression of Thy-1 (CD90) on acute myeloid leukemia cells with long-term proliferative ability in vitro and in vivo. Blood 89: 3104–3112. 9129012

[15] Hosen N, Park CY, Tatsumi N, Oji Y, Sugiyama H, et al (2007) CD96 is a leukemic stem cell-specific marker in human acute myeloid leukemia. Proc Natl Acad Sci U S A 104: 11008–11013. 17576927 10.1073/pnas.0704271104 PMC1904175

[16] Jan M, Chao MP, Cha AC, Alizadeh AA, Gentles AJ, et al (2011) Prospective separation of normal and leukemic stem cells based on differential expression of TIM3, a human acute myeloid leukemia stem cell marker. Proc Natl Acad Sci U S A 108: 5009–5014. 21383193 10.1073/pnas.1100551108 PMC3064328

[17] Jordan CT, Upchurch D, Szilvassy SJ, Guzman ML, Howard DS, et al (2000) The interleukin-3 receptor alpha chain is a unique marker for human acute myelogenous leukemia stem cells. Leukemia 14: 1777–1784. 11021753 10.1038/sj.leu.2401903

[18] Kikushige Y, Akashi K (2012) TIM-3 as a therapeutic target for malignant stem cells in acute myelogenous leukemia. Ann N Y Acad Sci 1266: 118–123. 22901263 10.1111/j.1749-6632.2012.06550.x

[19] Saito Y, Kitamura H, Hijikata A, Tomizawa-Murasawa M, Tanaka S, et al (2010) Identification of therapeutic targets for quiescent, chemotherapy-resistant human leukemia stem cells. Sci Transl Med 2: 17ra19. 10.1126/scitranslmed.3000349 PMC3005290 20371479

[20] van Rhenen A, van Dongen GA, Kelder A, Rombouts EJ, Feller N, et al (2007) The novel AML stem cell associated antigen CLL-1 aids in discrimination between normal and leukemic stem cells. Blood 110: 2659–2666. 17609428 10.1182/blood-2007-03-083048

[21] Thiede C, Steudel C, Mohr B, Schaich M, Schakel U, et al (2002) Analysis of FLT3-activating mutations in 979 patients with acute myelogenous leukemia: association with FAB subtypes and identification of subgroups with poor prognosis. Blood 99: 4326–4335. 12036858 10.1182/blood.v99.12.4326

[22] Thiede C, Koch S, Creutzig E, Steudel C, Illmer T, et al (2006) Prevalence and prognostic impact of NPM1 mutations in 1485 adult patients with acute myeloid leukemia (AML). Blood 107: 4011–4020. 16455956 10.1182/blood-2005-08-3167

[23] Waskow C, Liu K, Darrasse-Jeze G, Guermonprez P, Ginhoux F, et al (2008) The receptor tyrosine kinase Flt3 is required for dendritic cell development in peripheral lymphoid tissues. Nat Immunol 9: 676–683. 18469816 10.1038/ni.1615 PMC2746085

[24] Thiede C, Creutzig E, Illmer T, Schaich M, Heise V, et al (2006) Rapid and sensitive typing of NPM1 mutations using LNA-mediated PCR clamping. Leukemia 20: 1897–1899. 17041639 10.1038/sj.leu.2404367

[25] Bueno C, Montes R, de la Cueva T, Gutierrez-Aranda I, Menendez P (2010) Intra-bone marrow transplantation of human CD34(+) cells into NOD/LtSz-scid IL-2rgamma(null) mice permits multilineage engraftment without previous irradiation. Cytotherapy 12: 45–49. 19929453 10.3109/14653240903377052

[26] Bruggemann M, White H, Gaulard P, Garcia-Sanz R, Gameiro P, et al (2007) Powerful strategy for polymerase chain reaction-based clonality assessment in T-cell malignancies Report of the BIOMED-2 Concerted Action BHM4 CT98–3936. Leukemia 21: 215–221. 17170730 10.1038/sj.leu.2404481

[27] Du JW, Gu JY, Liu J, Cen XN, Zhang Y, et al (2007) TCR spectratyping revealed T lymphocytes associated with graft-versus-host disease after allogeneic hematopoietic stem cell transplantation. Leuk Lymphoma 48: 1618–1627. 17701594 10.1080/10428190701474357

[28] Gorin NC, Piantadosi S, Stull M, Bonte H, Wingard JR, et al (2002) Increased risk of lethal graft-versus-host disease-like syndrome after transplantation into NOD/SCID mice of human mobilized peripheral blood stem cells, as compared to bone marrow or cord blood. J Hematother Stem Cell Res 11: 277–292. 11983099 10.1089/152581602753658466

[29] Gregoire-Gauthier J, Durrieu L, Duval A, Fontaine F, Dieng MM, et al (2012) Use of immunoglobulins in the prevention of GvHD in a xenogeneic NOD/SCID/gammac- mouse model. Bone Marrow Transplant 47: 439–450. 21572464 10.1038/bmt.2011.93

[30] Hesselton RM, Greiner DL, Mordes JP, Rajan TV, Sullivan JL, et al (1995) High levels of human peripheral blood mononuclear cell engraftment and enhanced susceptibility to human immunodeficiency virus type 1 infection in NOD/LtSz-scid/scid mice. J Infect Dis 172: 974–982. 7561218 10.1093/infdis/172.4.974

[31] King MA, Covassin L, Brehm MA, Racki W, Pearson T, et al (2009) Human peripheral blood leucocyte non-obese diabetic-severe combined immunodeficiency interleukin-2 receptor gamma chain gene mouse model of xenogeneic graft-versus-host-like disease and the role of host major histocompatibility complex. Clin Exp Immunol 157: 104–118. 19659776 10.1111/j.1365-2249.2009.03933.x PMC2710598

[32] Ramirez M, Rottman GA, Shultz LD, Civin CI (1998) Mature human hematopoietic cells in donor bone marrow complicate interpretation of stem/progenitor cell assays in xenogeneic hematopoietic chimeras. Exp Hematol 26: 332–344. 9546317

[33] van Rijn RS, Simonetti ER, Hagenbeek A, Hogenes MC, de Weger RA, et al (2003) A new xenograft model for graft-versus-host disease by intravenous transfer of human peripheral blood mononuclear cells in RAG2−/− gammac−/− double-mutant mice. Blood 102: 2522–2531. 12791667 10.1182/blood-2002-10-3241

[34] Verlinden SF, Mulder AH, de Leeuw JP, van Bekkum DW (1998) T lymphocytes determine the development of xeno GVHD and of human hemopoiesis in NOD/SCID mice following human umbilical cord blood transplantation. Stem Cells 16 Suppl 1205–217. 11012164 10.1002/stem.5530160825

[35] Paz Morante M, Briones J, Canto E, Sabzevari H, Martino R, et al (2006) Activation-associated phenotype of CD3 T cells in acute graft-versus-host disease. Clin Exp Immunol 145: 36–43. 16792671 10.1111/j.1365-2249.2006.03104.x PMC1942002

[36] King M, Pearson T, Shultz LD, Leif J, Bottino R, et al (2008) A new Hu-PBL model for the study of human islet alloreactivity based on NOD-scid mice bearing a targeted mutation in the IL-2 receptor gamma chain gene. Clin Immunol 126: 303–314. 18096436 10.1016/j.clim.2007.11.001

[37] Cozzio A, Passegue E, Ayton PM, Karsunky H, Cleary ML, et al (2003) Similar MLL-associated leukemias arising from self-renewing stem cells and short-lived myeloid progenitors. Genes Dev 17: 3029–3035. 14701873 10.1101/gad.1143403 PMC305255

[38] Haase D, Feuring-Buske M, Konemann S, Fonatsch C, Troff C, et al (1995) Evidence for malignant transformation in acute myeloid leukemia at the level of early hematopoietic stem cells by cytogenetic analysis of CD34+ subpopulations. Blood 86: 2906–2912. 7579382

[39] Hanahan D, Weinberg RA (2011) Hallmarks of cancer: the next generation. Cell 144: 646–674. 21376230 10.1016/j.cell.2011.02.013

[40] So CW, Karsunky H, Passegue E, Cozzio A, Weissman IL, et al (2003) MLL-GAS7 transforms multipotent hematopoietic progenitors and induces mixed lineage leukemias in mice. Cancer Cell 3: 161–171. 12620410 10.1016/s1535-6108(03)00019-9

[41] Martelli MP, Manes N, Pettirossi V, Liso A, Pacini R, et al (2008) Absence of nucleophosmin leukaemic mutants in B and T cells from AML with NPM1 mutations: implications for the cell of origin of NPMc+ AML. Leukemia 22: 195–198. 17637812 10.1038/sj.leu.2404857

[42] Pasqualucci L, Liso A, Martelli MP, Bolli N, Pacini R, et al (2006) Mutated nucleophosmin detects clonal multilineage involvement in acute myeloid leukemia: Impact on WHO classification. Blood 108: 4146–4155. 16926285 10.1182/blood-2006-06-026716

[43] Agliano A, Martin-Padura I, Mancuso P, Marighetti P, Rabascio C, et al (2008) Human acute leukemia cells injected in NOD/LtSz-scid/IL-2Rgamma null mice generate a faster and more efficient disease compared to other NOD/scid-related strains. Int J Cancer 123: 2222–2227. 18688847 10.1002/ijc.23772

[44] Malaise M, Neumeier M, Botteron C, Dohner K, Reinhardt D, et al (2011) Stable and reproducible engraftment of primary adult and pediatric acute myeloid leukemia in NSG mice. Leukemia 25: 1635–1639. 21647161 10.1038/leu.2011.121

[45] Sanchez PV, Perry RL, Sarry JE, Perl AE, Murphy K, et al (2009) A robust xenotransplantation model for acute myeloid leukemia. Leukemia 23: 2109–2117. 19626050 10.1038/leu.2009.143 PMC3659827

[46] Ailles LE, Gerhard B, Kawagoe H, Hogge DE (1999) Growth characteristics of acute myelogenous leukemia progenitors that initiate malignant hematopoiesis in nonobese diabetic/severe combined immunodeficient mice. Blood 94: 1761–1772. 10477702

[47] Bonnet D, Dick JE (1997) Human acute myeloid leukemia is organized as a hierarchy that originates from a primitive hematopoietic cell. Nat Med 3: 730–737. 9212098 10.1038/nm0797-730

[48] Lapidot T, Sirard C, Vormoor J, Murdoch B, Hoang T, et al (1994) A cell initiating human acute myeloid leukaemia after transplantation into SCID mice. Nature 367: 645–648. 7509044 10.1038/367645a0

[49] Rombouts WJ, Martens AC, Ploemacher RE (2000) Identification of variables determining the engraftment potential of human acute myeloid leukemia in the immunodeficient NOD/SCID human chimera model. Leukemia 14: 889–897. 10803522 10.1038/sj.leu.2401777

[50] Risueno RM, Campbell CJ, Dingwall S, Levadoux-Martin M, Leber B, et al (2011) Identification of T-lymphocytic leukemia-initiating stem cells residing in a small subset of patients with acute myeloid leukemic disease. Blood 117: 7112–7120. 21562049 10.1182/blood-2011-01-329078

[51] Ali N, Flutter B, Sanchez Rodriguez R, Sharif-Paghaleh E, Barber LD, et al (2012) Xenogeneic graft-versus-host-disease in NOD-scid IL-2Rgammanull mice display a T-effector memory phenotype. PLoS One 7: e44219. 22937164 10.1371/journal.pone.0044219 PMC3429415

[52] Greenblatt MB, Vbranac V, Tivey T, Tsang K, Tager AM, et al (2012) Graft versus host disease in the bone marrow, liver and thymus humanized mouse model. PLoS One 7: e44664. 22957096 10.1371/journal.pone.0044664 PMC3434179

[53] Ishikawa F, Yoshida S, Saito Y, Hijikata A, Kitamura H, et al (2007) Chemotherapy-resistant human AML stem cells home to and engraft within the bone-marrow endosteal region. Nat Biotechnol 25: 1315–1321. 17952057 10.1038/nbt1350

[54] Martin MG, Welch JS, Uy GL, Fehniger TA, Kulkarni S, et al (2010) Limited engraftment of low-risk myelodysplastic syndrome cells in NOD/SCID gamma-C chain knockout mice. Leukemia 24: 1662–1664. 20668474 10.1038/leu.2010.156 PMC3341627

[55] Patel S, Zhang Y, Cassinat B, Zassadowski F, Ferre N, et al (2012) Successful xenografts of AML3 samples in immunodeficient NOD/shi-SCID IL2Rgamma(-)/(-) mice. Leukemia 26: 2432–2435. 22699451 10.1038/leu.2012.154

[56] Yoshida S, Ishikawa F, Yasukawa M, Miyamoto T, Yoshimoto G, et al (2005) Long-term engraftment and self-renewal of AML stem cells in the newborn NOD-scid/IL2rgmll immumodeficient mouse model. Blood 106: 367A–367A.

[57] Bagnara D, Kaufman MS, Calissano C, Marsilio S, Patten PE, et al (2011) A novel adoptive transfer model of chronic lymphocytic leukemia suggests a key role for T lymphocytes in the disease. Blood 117: 5463–5472. 21385850 10.1182/blood-2010-12-324210 PMC3109718

