# Supplementary figures and images for: In Vivo Expansion of Co-Transplanted T Cells Impacts on Tumor Re-Initiating Activity of Human Acute Myeloid Leukemia in NSG Mice
Source: PLoS One. 2013 Apr 9;8(4):e60680. doi: 10.1371/journal.pone.0060680 (PMC3621959; doi:10.1371/journal.pone.0060680)

# Supplementary Figure 1

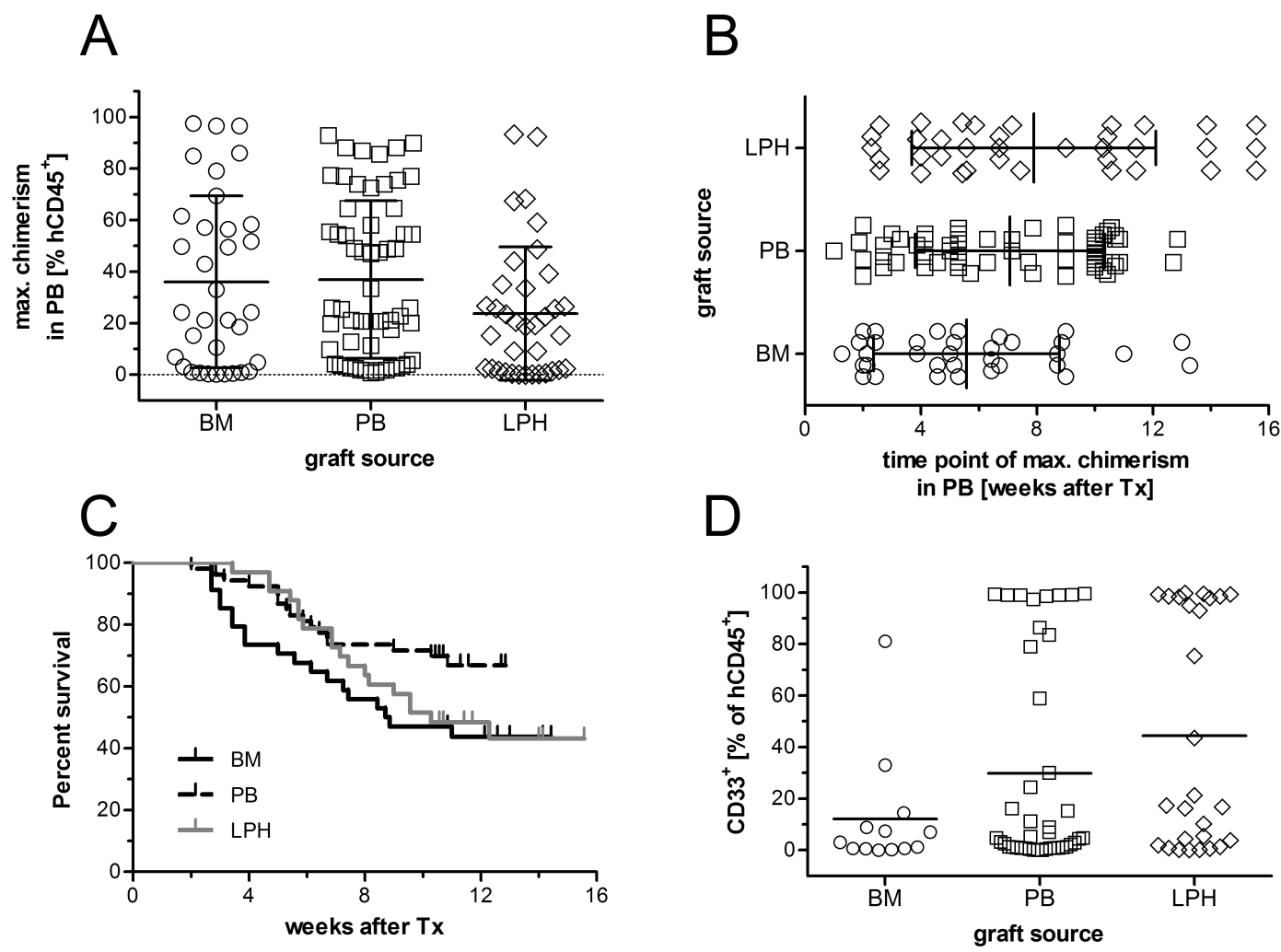

Supplement: Figure S1 — Source of AML-MNCs influences the engraftment kinetics of human leukocytes. MNCs from patients with AML were isolated from bone marrow (BM), peripheral blood (PB) or leukapheresis products (LPH), and 5×10^6^–107 MNCs were transplanted freshly or after DMSO-based storage into non-conditioned or irradiated NSG mice. Recipient mice were sacrificed 12–16 weeks after transplantation or earlier when detoriation of health occurred. (A) The maximum human leukocyte chimerism in the blood during the observation period is presented in dependency of the graft source. (B) The graph shows the time point of the maximum peripheral blood chimerism in mice that received MNCs from bone marrow, blood or LPH samples from AML patients with AML. (C) Survival of mice transplanted with MNCs from bone marrow, blood or LPH samples from AML patients. (D) Plot depicts the frequency of human myeloid cells (CD33+) within all engrafted human leukocytes (hCD45+) according to the sample source. (PDF) [file pone.0060680.s001.pdf]

## Supplementary Figure 2

A

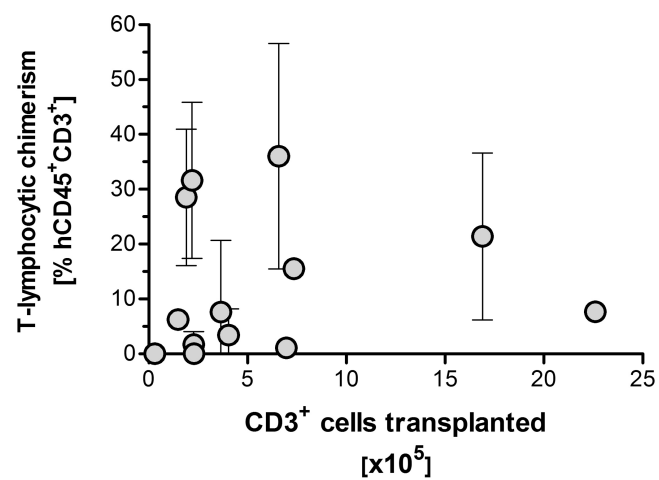

B

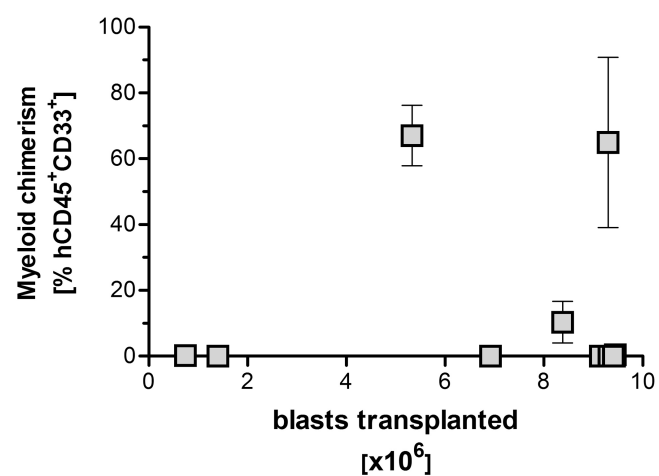

C

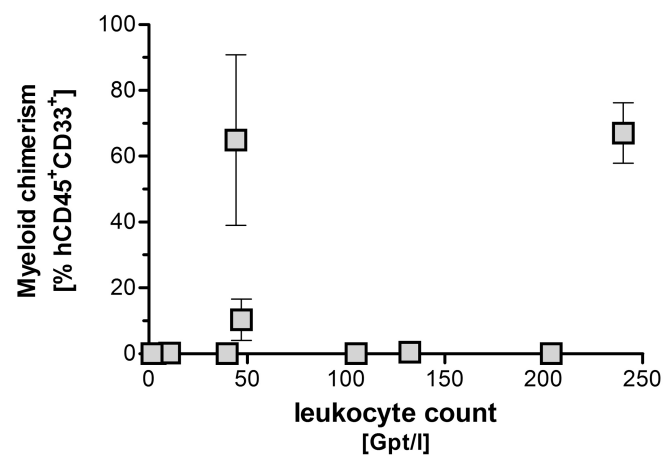

Supplement: Figure S2 — Lack of correlation between the type of engraftment and the cellular composition of the graft. Frequencies of hCD45+ CD3+ (A) or hCD45+ CD33+ (B, C) positive cells in the bone marrow of NSG mice that were transplanted with 5×10^6^–107 freshly isolated MNCs from 13 (A) or 11 (B+C) AML patients before. Frequency of CD3+ donor cells is depicted as a function of CD3+ cells in the graft (A). Frequency of CD33+ donor cells is depicted as a function of the number of AML blasts in the graft (side scatterlow CD45+, B) or total leukocytes of the patient (C). (PDF) [file pone.0060680.s002.pdf]

Supplementary Figure 3

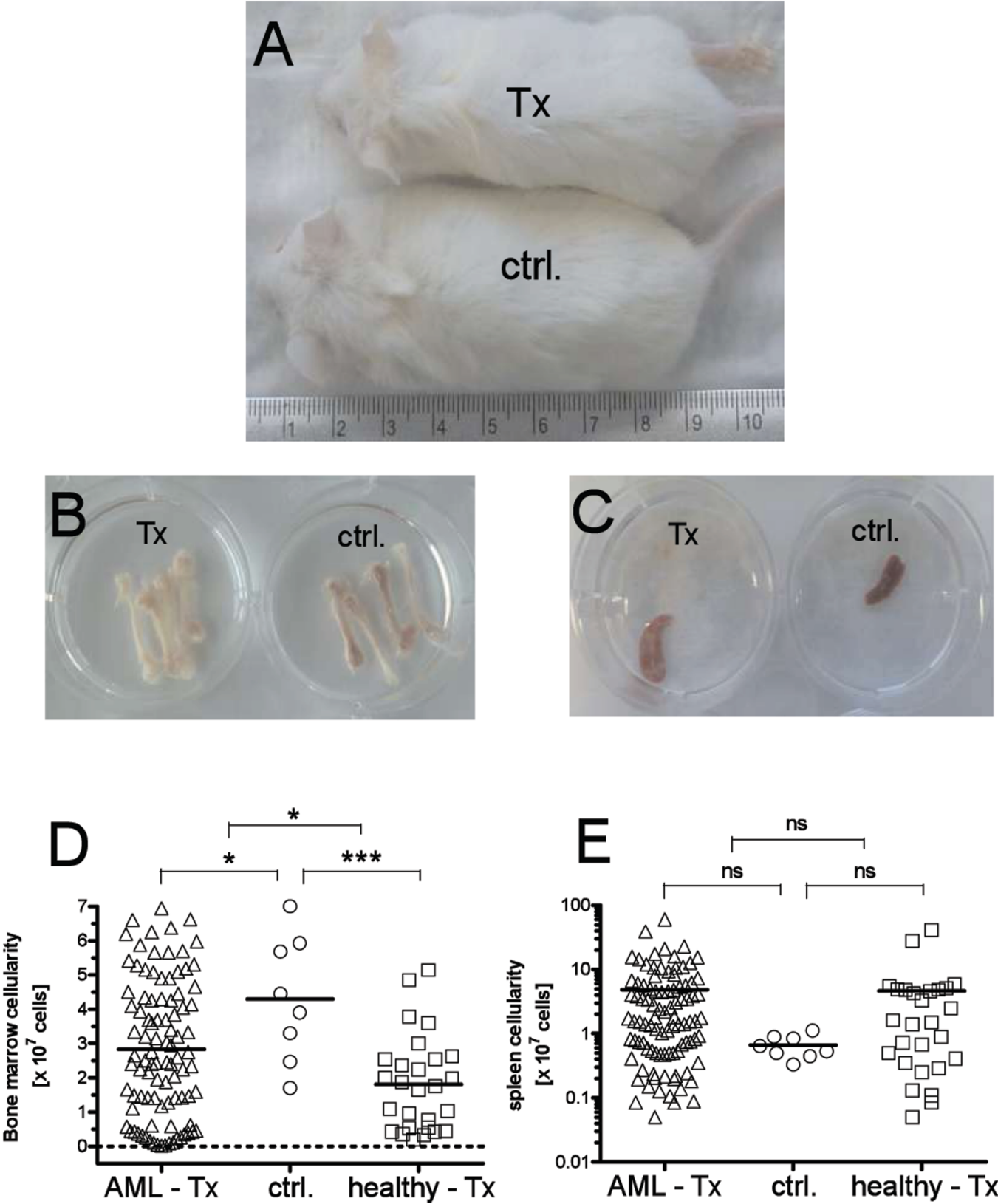

Supplement: Figure S3 — Xenogeneic graft versus host disease leads to growth retardation, splenomegaly and bone marrow hypoplasia. (A) Picture shows NSG recipient mice that were transplanted 107 MNCs (PB) from a healthy donor 6 weeks before (Tx) and non-transplanted control mice (ctrl.). Human donor-cell chimerism in the peripheral blood, bone marrow and spleen was 32%, 19% and 86%, respectively, and in all organs >98% of all human leukocytes expressed the CD3 antigen. Picture is representative for 5 independent recipient mice. (B) Femuras and tibias of mice that had received MNCs from healthy individuals as described in (A) were pale compared to control NSG mouse bones. (C) The spleen of mice that had received MNCs from healthy individuals was enlarged compared to a control spleen from a non-injected NSG mouse. Spleens depicted originate from mice described in (A). (D) Plot shows reduced bone marrow cellularity in mice that were transplanted with 5×10^6^–107 MNCs from patients (left) or healthy donors (right). Bone marrow hypoplasia is detected in mice transplanted with MNCs from healthy individuals (1.8±1.4×10^7^) and from AML patients (2.8±2.0×10^7^), compared to non-transplanted NSG mice (middle, 4.3±1.8×10^7^). (E) Mean spleen cellularity of mice transplanted with MNCs of healthy donors (right, 4.6±9.1×10^7^) or AML patients (left, 4.9±8.4×10^7^) or untreated NSG mice (middle, 0.7±2.7×10^7^). Mice received grafts described in (D). (PDF) [file pone.0060680.s003.pdf]
