# Supplementary material for: In Vivo Expansion of Co-Transplanted T Cells Impacts on Tumor Re-Initiating Activity of Human Acute Myeloid Leukemia in NSG Mice
Source: PLoS One. 2013 Apr 9;8(4):e60680. doi: 10.1371/journal.pone.0060680 (PMC3621959; doi:10.1371/journal.pone.0060680)
Supplement: Figure S4 — Transplantation of MNCs from healthy donors results in almost exclusive engraftment of human T lymphocytes. Unconditioned NSG mice were transplanted with 5×10^6^–107 MNCs freshly isolated from the bone marrow and blood of healthy volunteers and leukapheresis products from G-CSF treated donors. Mice were sacrificed 12 weeks after transplantation or when status of health detoriated. (A) Mice transplanted with MNCs from healthy donors showed a significant shortened survival compared to mice transplanted with AML-MNCs. (B) Transplantation of MNCs of healthy donors led to organ specific chimerism that was determined at the time point of analysis. (C) Donor-derived leukocytes in the blood, BM and spleen of NSG mice that had received MNCs from healthy donors expressed predominantly CD3+ T lymphocytes, while CD19+ B-lymphocytes and CD33+ myeloid cells were barely detectable. (D) Plot shows the frequency of TCRα/β+ and TCRγ/δ+ T cells in CD3+ cells, and the frequency of the expression of CD4 and CD8 or both on TCRα/β+ T cells on donor-derived lymphocytes in the bone marrow of NSG recipient mice after the transplantation of MNCs from healthy donors. (CD4∶52.2±15.7%, CD8∶34.9±14.1%, CD4/CD8∶10.5±5.5%, TCRα/β: 97.3±9.5% and TCRγ/δ: 0.09±0.2%) (E) Plot shows the frequency of indicated Vβ segments in human α/β T cell receptors on T cells in the bone marrow of NSG mice that had received MNCs from healthy volunteers. Frequencies based on human CD45+ CD3+ cells are shown. (F) Bar graph shows the frequency of human T lymphocytes (hCD45+CD3+) that express CD25 and/or CD69 in blood, bone marrow and spleen of NSG recipient mice that were transplanted with MNCs from healthy patients. N = 26 for all groups. (PDF) [file pone.0060680.s004.pdf]

Supplementary Figure 4

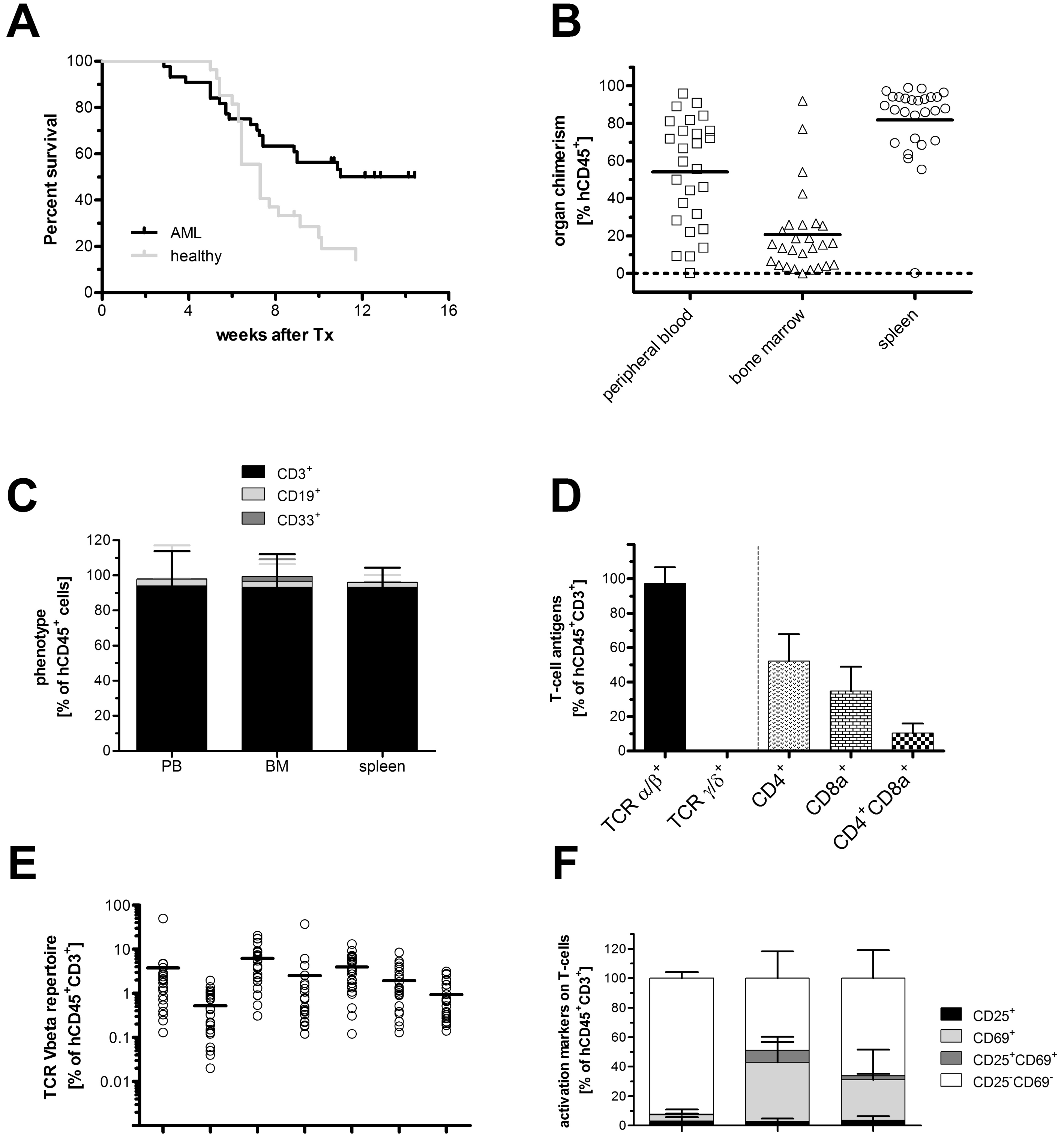

	peripheral blood	bone marrow	spleen
$CD25^+$	3.1±2.7	2.9±1.9	3.5±2.8
$CD69^+$	4.2±3.7	40.1±17.2	27.6±20.4
$CD25^+CD59^+$	0.4±0.4	8.1±5.7	2.6±1.5
$CD25^-CD69^-$	92.3±4.3	48.9±18.1	66.3±19.0
